# Primary hypertrophic osteoarthropathy complicated with myelofibrosis and compound heterozygous SLCO2A1 mutations: a case report and review of literature

**DOI:** 10.3389/fonc.2026.1772374

**Published:** 2026-06-30

**Authors:** Qirui Xu, Qian Li, Jin Lu, Liru Wang

**Affiliations:** 1 Department of Hematology, Fu Xing Hospital, Capital Medical University, Beijing, China; 2 Peking University People’s Hospital, National Clinical Research Center for Hematologic Diseases, Peking University Institute of Hematology, Beijing, China

**Keywords:** biallellic variants, compound heterozygous mutation, myelofibrosis, primary hypertrophic osteoarthropathy, SLCO2A1

## Abstract

**Background:**

Primary hypertrophic osteoarthropathy (PHO) is a rare hereditary clinical syndrome characterized by digital clubbing, periostosis, and pachydermia. It is mainly caused by mutations in SLCO2A1 or HPGD, leading to impaired degradation and elevated levels of prostaglandin E2 (PGE2). In addition to the typical skeletal and skin manifestations, some patients may present with gastrointestinal or hematologic abnormalities, including anemia and myelofibrosis. This report describes a case of PHO with myelofibrosis and compound heterozygous SLCO2A1 mutations and review the literature.

**Case report:**

A 45-year-old man presented with a long history of severe fatigue and recurrent anemia accompanied by digital clubbing and skin thickening. Bone marrow biopsy revealed myelofibrosis, and imaging studies showed periostosis of the long bones. Given the anemia and gastrointestinal symptoms, differential diagnoses included Crohn’s disease, intestinal tuberculosis, and other hematologic disorders associated with myelofibrosis, which were excluded by endoscopic and hematologic evaluations. Whole-exome sequencing revealed that the patient carried compound heterozygous variants in SLCO2A1 (c.940 + 1G>A and c.440G>A/p.Trp147*), confirming the diagnosis of PHO. Family investigation showed that his parents and offspring were all asymptomatic carriers with one heterozygous variant, which is consistent with autosomal recessive inheritance.

**Conclusion:**

Myelofibrosis is a rare but important complication of PHO and may be more frequent in patients with biallelic SLCO2A1 variants. Genetic testing and hematologic evaluation in PHO patients can help with early identification of related complications.

## Introduction

Primary hypertrophic osteoarthropathy (PHO), also known as pachydermoperiostosis (PDP, MIM 259100, 614441, 167100), is a hereditary clinical syndrome with complex manifestations. The disease was first described by Friedreich in 1868, characterized by Touraine in 1935 and later classified by Harbinson in 1971 (1). PHO is a rare disorder, accounting for approximately 3-5% of all cases of all hypertrophic osteoarthropathy, with a higher prevalence reported in East Asian populations and a marked male predominance (2, 3). Its precise incidence remains unknown, and most data come from sporadic case reports rather than population-based studies.

The pathogenesis is closely associated with abnormalities in the prostaglandin metabolic pathway, particularly the sustained elevation of prostaglandin E2 (PGE2) levels. Two causative genes have been identified: SLCO2A1 (MIM 601460) and HPGD (MIM 601688). Mutations in either gene result in increased local concentrations of PGE2, which is thought to lead to the main clinical features of the disease, including digital clubbing, pachydermia, and periosteal proliferation. PHO is mostly inherited in autosomal recessive manner, which makes the identification of pathogenic variants particularly important for confirming the diagnosis and providing genetic counseling.

In addition to these skeletal and cutaneous features, hematological abnormalities such as anemia and myelofibrosis have also been reported. With more than 500 cases of PHO reported, only 22 cases complicated by myelofibrosis have been documented to date, highlighting the extreme rarity of this condition (4). These manifestations pose considerable challenges for the differential diagnosis of patients with overlapping skeletal, cutaneous and hematologic abnormalities, underscore the importance of recognizing PHO as a potential cause of secondary myelofibrosis, and expand the genotypic and phenotypic spectrum of SLCO2A1-related disease. Here, we present a case of PHO with myelofibrosis and compound heterozygous SLCO2A1 mutations and review related literature.

## Case report

**Figure 1:**
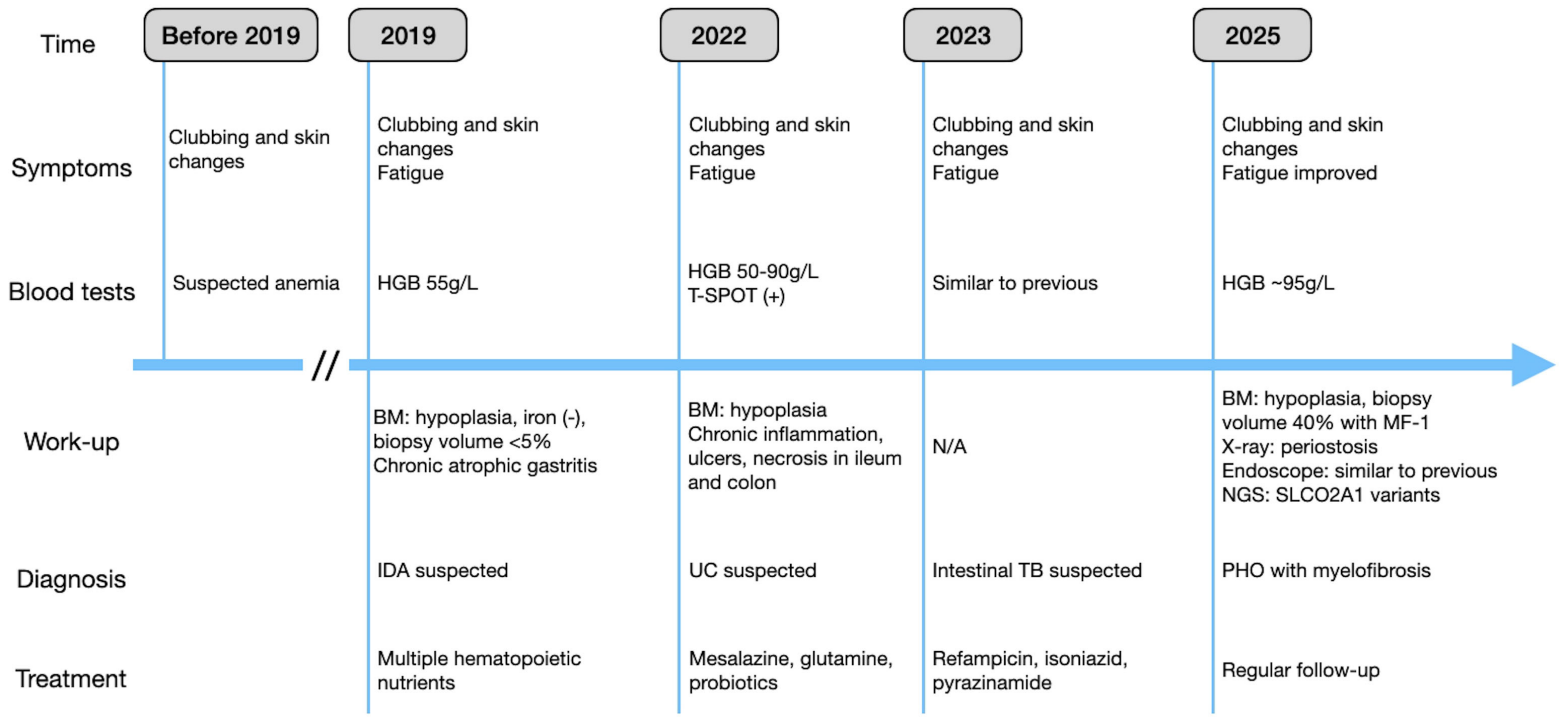
Timeline of the patient’s clinical journey. HGB, hemoglobin; IDA, iron defeciency anemia; UC, ulcerative colitis; TB, tubercolosis; N/A, not applicable or not available; NGS, next-generation-sequencing.

**Figure 2:**
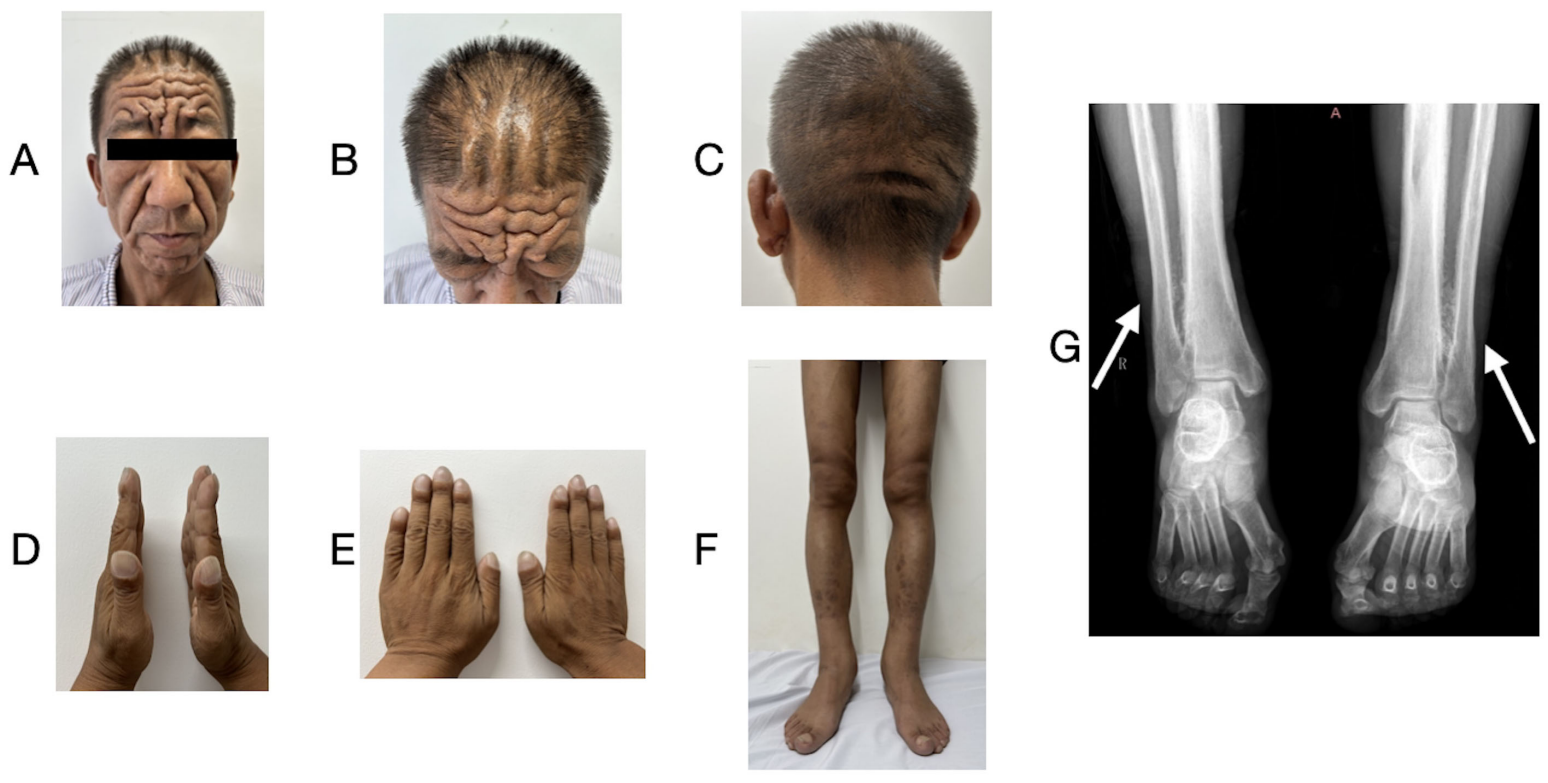
Clinical manifestations of the proband. **(A-C)** Pachydermia with prominent folds on the front, top and back of the head. **(D, E)** Digital clubbing. **(F)** Knee and ankle arthropathy and columnar swelling of the lower limbs. **(G)** X-ray showing periostosis of the lower limbs.

The patient was a 45-year-old male who had sought medical attention at multiple hospitals nationwide over the years with no definitive diagnosis established. His chief complaint was severe fatigue and decreased hemoglobin levels for over 6 years, first documented in 2019, although chronic anemia was suspected to have been present for a longer period of time without systemic evaluation or treatment. He reported occasional night sweats, but denied fever, abdominal pain, diarrhea, or other specific symptoms. He reported a remote history of childhood tuberculosis which had been cured with regular anti-tuberculosis therapy. He also reported a long-term smoking history and a history of blood transfusion during previous treatments without adverse reactions. The patient denied other prior medical conditions or family history of such symptoms or related disorders. On physical examination, marked digital clubbing and skin thickening with prominent folds on the forehead, face, and scalp was observed. The patient reported that these changes began around the age of 18, gradually worsening but without affecting function. The lower limb joints including the knees and ankles appeared slightly swelled. The spleen was not palpable. A timeline summarizing the patient’s clinical journey is shown in **Figure 1**. The patient’s clinical manifestations are presented in **Figure 2**.

This study was approved by Ethics Review Committee of Fu Xing Hospital. Written informed consent for publication was obtained from the patient.

### Blood tests

In June 2019, the complete blood count (CBC) revealed leukocyte 4.52×10^9^/L, hemoglobin 55g/L and platelet 154×10^9^/L, with MCV and MCHC within normal range. Serum vitamin B12 (928.20pg/ml) and folate (>20ng/mL) was normal, while elevated serum ferritin (468. 59ng/mL) and EPO (>750mlU/mL) was noted. Other tests including auto antibodies, hepatitis antibodies, thyroid function, growth hormone and tumor markers were within normal limits. The tests in 2022 showed comparable results, leukocyte 5.08×10^9^/L, hemoglobin 52g/L, platelet 152×10^9^/L, MCV 95.4fl, MCHC 315g/L, reticulocyte 35.80×10^9^/L. Erythrocyte sedimentation rate was 100ml/h. Anti-tuberculosis antibody was negative, whereas the T-SPOT.TB test was positive. Upon admission in 2025, the CBC revealed a hemoglobin level of approximately 95g/L. Fecal occult blood testing was positive. Other laboratory results were entirely consistent with the previous results.

### Bone marrow examinations

In 2019, bone marrow aspiration of iliac crest demonstrated significant hypoplasia with biopsy showing hematopoietic volume less than 5% (silver staining was not performed), while another aspiration of sternum showed normal hyperplasia of erythrocytes and absence of storage iron and sideroblasts. The bone marrow aspiration in 2022 showed comparable hypoplasia with no other significant findings. In 2025, bone marrow aspiration demonstrated a slight hypoplasia with active erythroid proliferation. Overall, flow cytometry demonstrated largely preserved hematopoietic maturation without evidence of an abnormal blast population or aberrant immunophenotype. Minor variations in subset proportions were observed, including reduced lymphocytes and B-cell precursors and a relative increase in CD11b^−^ granulocytes. Biopsy revealed a hematopoietic cellularity of approximately 40% with MF-1 reticulin fibrosis, while the marrow demonstrated active proliferation with a reduced granulocyte-to-erythroid ratio and an increased number of megakaryocytes. Multiplex RT-PCR was performed to screen for JAK2 V617F and 42 recurrent leukemia-associated fusion genes (including BCR::ABL1, PML::RARA, RUNX1::RUNX1T1, CBFB::MYH11, KMT2A rearrangements, and ETV6::RUNX1, among others), and all results were negative.

### Image studies

Previous CT examinations were reported to show heterogeneous bone density involving the ribs, sternum, and thoracic vertebrae. The original reports were unavailable. During the current presentation, X-ray examination of limbs showed marked periostosis (**Figure 2**).

### Endoscopy findings

In 2019, gastroscope indicated anemic gastric mucosal changes and chronic atrophic gastritis with hyperplasia and erosion. In 2022, colonoscope demonstrated ulcers in the terminal ileum and ileocecal region. Biopsy of the tissue showed chronic inflammation with ulceration and necrotic exudates, consistent with ileocecal ulcer. The endoscope and biopsy during the current presentation in 2025 showed chronic active inflammation accompanied by ulcer formation and granulation tissue hyperplasia involving ileocecal valve, ascending colon and hepatic flexure, which is comparable to the previous results.

### Therapeutic management

In 2019, the patient was first diagnosed with iron deficiency anemia and received supplementation with multiple hematopoietic nutrients including iron. The response was ineffective and hemoglobin levels remained fluctuating around 50-90g/L. In 2022, based on a presumptive diagnosis of ulcerative colitis, the patient was treated with mesalazine, glutamine and probiotics for approximately one year. There was still no improvement in fatigue or hemoglobin levels during or after the therapy. Subsequently, the patient was transferred to another local hospital, where a trial therapy for intestinal tuberculosis was initiated. He received a combined regimen of refampicin, isoniazid and pyrazinamide for about one year from January 2023 to January 2024. The fatigue and anemia persisted with no improvements, with hemoglobin levels fluctuating around 60-90g/L.

### Genetic testing

To identify the genetic basis of the disease, whole exome sequencing (WES) was performed on the proband after obtaining informed consent. Peripheral blood samples were collected from the proband and family members into EDTA tubes. Genomic DNA was extracted using a magnetic bead-based kit (Tiangen, DP705), and quality was assessed by agarose gel electrophoresis and Qubit quantification. Samples with ≥0.6 µg DNA were used for library preparation. Whole-exome capture was performed using the IDT ×Gen Exome Research Panel v1.0 (39 Mb) and sequenced on a GeneMind SURFseq5000 platform. The assay was designed to detect single nucleotide variants (SNVs), small insertions/deletions (indels <50 bp), and variants located within exonic regions and flanking intronic sequences (± 20 bp). Sequencing quality metrics met the laboratory standards, with an average target-region sequencing depth of approximately 100×, Q30 ≥85%, and Q20 ≥90%. Raw sequencing data underwent quality control and were aligned to the human reference genome (GRCh37/hg19), followed by variant calling and annotation using a standardized bioinformatics pipeline provided by the certified testing laboratory. Variant interpretation was performed using multiple population and disease databases, including dbSNP, ClinVar, gnomAD, RefSeq, Chinese Human Phenotype Ontology (CHPO), etc. Pathogenicity was evaluated according to the ACMG guidelines, with functional prediction tools such as REVEL used as supporting evidence.

The analysis revealed that the patient carried compound heterozygous variants of SLCO2A1 (NM_005630.3), c.940 + 1G>A (39x reads, depth 86x) and c.440G>A/p.Trp147* (88x reads, depth 167x). According to ACMG guidelines, both variants in SLCO2A1 were classified as pathogenic (PVS1, PM3_Very Strong, PM2_Supporting and PVS1, PM3_Strong, PM2_Supporting, respectively). According to gnomAD database, the minor allele frequency (MAF) of c.940 + 1G>A in the East Asian population was 0.00033, whereas c.440G>A/p.Trp147* was absent from population databases.

**Figure 3:**
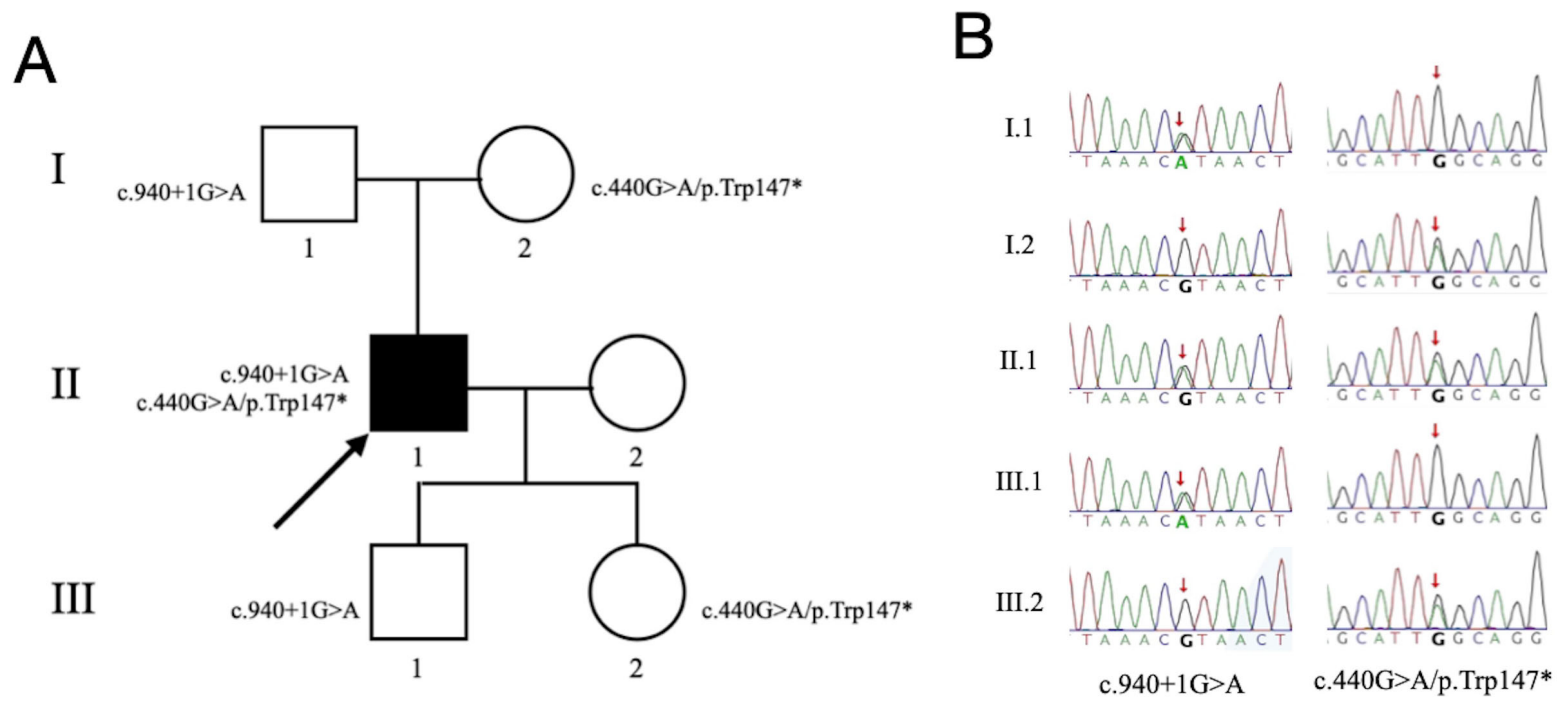
Pedigree analysis and Sanger sequencing validation of the SLCO2A1 variants. **(A)** Pedigree of the family. All available family members underwent Sanger sequencing except II.2. Squares indicate males and circles indicate females. The proband (II.1) is indicated by an arrow. Affected individuals are indicated by filled symbols, unaffected individuals are indicated by open symbols. Variants are indicated next to each individual. The proband carried compound heterozygous variants of SLCO2A1 (NM_005630.3) c.940 + 1G>A and c.440G>A/p.Trp147*. Each parent and child was a heterozygous carrier, consistent with autosomal recessive inheritance. **(B)** Sanger sequencing validation of the variants.

Candidate variants were validated in available family members (the father, mother, two offspring and the proband) by Sanger sequencing using primers designed in Primer Premier 5.0 and purified PCR products sequenced on an ABI 3730XL. Segregation analysis showed that the parents of the proband each carried one heterozygous variant. Specifically, the father and the proband’s son carried the c.940 + 1G>A variant, while the mother and the proband’s daughter carried the c.440G>A (p.Trp147) variant. Both variants have been reported as pathogenic and are predicted to result in loss of function of SLCO2A1, which is consistent with the known molecular mechanism of PHO. Other family members carrying a single heterozygous variant were asymptomatic, showing no skin changes or anemia, whereas the proband inherited both variants and was the only affected individual in the family, a segregation pattern which is consistent with autosomal recessive inheritance. The details of family pedigree analysis is shown in **Figure 3**.

### Follow-up

After the diagnosis of PHO was established, the patient’s condition remained largely stable, with mild fatigue and no other symptoms such as impaired mobility, abdominal pain, or diarrhea. Hemoglobin levels remained stable above 90g/L. Due to physical, psychological and financial stress, the patient temporarily declined further treatment intervention to avoid additional medical visits. Selective COX-2 inhibitors were not administered due to the lack of efficacy in improving myelofibrosis and the potential risk of gastrointestinal bleeding. He was managed with regular follow-up, which has continued for approximately 8 months.

## Discussion

Primary hypertrophic osteoarthropathy is a rare hereditary bone disorder associated with abnormal prostaglandin metabolism, accounting for approximately 3%–5% of cases of hypertrophic osteoarthropathy. In 2008, Uppal et al. (5) first identified HPGD as a causative gene, which encodes 15-hydroxyprostaglandin dehydrogenase (15-PGDH), a key enzyme responsible for degrading PGE2 into inactive metabolites. Zhang et al. (6) reported another pathogenic gene, SLCO2A1, which encodes a prostaglandin transporter that facilitates the uptake of prostaglandin from extracellular space into the cell. There are currently two classifications of the disease. Based on the patterns of inheritance, PHO can be classified into three subtypes: autosomal recessive 1 (PHOAR1, MIM 259100), autosomal recessive 2 (PHOAR2, MIM 614441), and autosomal dominant (PHOAD, MIM 167100). PHOAR1 results from mutations in HPGD (MIM 601688), while PHOAR2 and PHOAD are associated with mutations in SLCO2A1 (MIM 601460) (2). Clinically, PHOAR1 usually manifests shortly after birth and shows no sex difference, whereas PHOAR2 and PHOAD typically present during adolescence, with the majority of affected individuals being male, suggesting sex hormones playing a role during the development of PHO. PHOAR2 typically presents with more severe bone, skin and joint symptoms as well as anemia, while PHOAR1 and PHOAD generally show milder clinical features. In most patients, the disease gradually stabilizes and becomes self-limiting in adulthood. Based on clinical and radiographic features, PHO can be classified into three subtypes: (1) complete form: characterized by digital clubbing, pachydermia, and periostosis; (2) incomplete form: similar to the complete form but lacking pachydermia; and (3) fruste form: characterized by pachydermia without, or with only minimal, periostosis (1). Additional features may include seborrhea, hyperhidrosis, diarrhea, anemia, and myelofibrosis.

Since anemia and myelofibrosis are rare manifestations of PHO, many reports do not include marrow examinations or lack detailed descriptions of marrow findings, which may lead to an underestimation of the true incidence of myelofibrosis in PHO patients. Most cases are reported from Asian countries, whereas reports from Western countries are relatively rare. From the perspective of sex and age, myelofibrosis frequently appears in male PHO patients, and the onset often occurs in adolescence or early adulthood (7). A Chinese study (3) summarizing 533 PHO patients found that only about 10% had bone-marrow or joint disease. Another Chinese cohort (8) showed that 10.9% (15/137) patients presented initially with anemia, but only 3.8% (5/133) of them had accompanying myelofibrosis. Patients with gastrointestinal involvement were more likely to have myelofibrosis (19.0%,4/21) than those without (0.9%,1/112), which may be related to that SLCO2A1 is also the causative gene for another group of disease, chronic enteropathy associated with SLCO2A1 (CEAS). CEAS primarily manifests as nonspecific small intestinal ulcers and may be accompanied by anemia, hypoalbuminemia, and abdominal symptoms such as pain, diarrhea, or gastrointestinal bleeding. CEAS predominantly affects females, whereas PHOAR2 almost exclusively affects males (2). There are some overlapping clinical manifestations between the two conditions. Our patient could be categorized within this spectrum with his anemia likely resulting from the combined effects of both myelofibrosis and intestinal blood loss. This may partially explain the observed changes in disease severity, with increasing age, self-limiting features of the disease, and stabilization of both contributing factors, the patient’s hemoglobin levels improved to some extent.

For similar reasons, the differential diagnosis in this patient was particularly challenging. First, regarding the etiology of anemia, the presence of fecal occult blood indicated a clear association with chronic intestinal blood loss. Although the patient’s multisystem manifestations might potentially affect hematopoietic substrates, current evaluations showed no evidence of deficiency, and previous treatments such as iron supplementation showed limited efficacy, suggesting that insufficient substrates were unlikely to be the primary cause of anemia. Bone marrow examination showed no dysplasia or blasts with preserved erythroid proliferation, thereby excluding conditions such as myelodysplastic syndrome or pure red cell anemia secondary to specific causes (e.g., anti-tuberculosis therapy). Second, in the differential diagnosis of Intestinal manifestations, the patient lacked the typical systemic and intestinal symptoms of ulcerative colitis or intestinal tuberculosis. The pathological findings were not strongly suggestive of either condition. Under these circumstances, the patient’s history of tuberculosis was highly misleading. The poor response of empirical therapy helped to rule out both ulcerative clolitis and tuberculosis. Third, regarding myelofibrosis, heterogeneity in the degree of fibrosis and hematopoietic cellularity across different anatomical sites may account for the inconsistency observed in the patient’s findings across the years. Negative results for driver mutations could effectively exclude the majority of primary myelofibrosis, suggesting the myelofibrosis in this patient was likely secondary. Moreover, it is critical to pay attention to the patient’s overall features, as a single symptom may represent only a secondary manifestation of a comprehensive multisystem disorder. In this case, the long disease course and prominent skin abnormalities were important clues prompting consideration of a genetic etiology.

Myelofibrosis in PHO results from the combined effects of multiple pathways, together with additional genetic, hormonal, and inflammatory factors (2). Experimental studies suggest that prostaglandin E2 (PGE2) enhances hematopoietic stem cell survival, homing, and adhesion within the bone marrow niche, and promotes their self-renewal. Chronic elevation of PGE2 may disturb normal hematopoiesis, leading to megakaryocyte activation and increased secretion of cytokines, thereby creating conditions favorable for fibrosis (9). Moreover, PGE2 can stimulate VEGF expression, which promotes angiogenesis and increases vascular permeability within the marrow, and abnormal angiogenesis is thought to play an important role in the development of fibrosis (10). PGE2 also induces megakaryocyte activation and promotes PDGF release, driving fibroblast proliferation and extracellular matrix deposition (11). In any case, the pathogenesis of myelofibrosis in PHO patients’ needs to be further clarified in future studies.

Previous studies have indicated that in PHO patients, myelofibrosis occurs exclusively in those with SLCO2A1 mutations, whereas no such cases have been reported in patients carrying HPGD mutations (12). This trend was consistent with the difference between causative genes, as PHOAR2 is more likely to involve systemic manifestations, including gastrointestinal and hematologic abnormalities (2). To date, no clear genotype–phenotype correlation has been established between specific SLCO2A1 mutation sites or variant types and the development of myelofibrosis. Although the two variants identified in this patient (c.940 + 1G>A and c.440G>A/p.Trp147*) have been reported previously, this specific compound heterozygous combination has not been described. The c.940 + 1G>A variant is a splice-site variant that is predicted to disrupt normal mRNA splicing, whereas the c.440G>A (p.Trp147*) variant is a nonsense mutation predicted to result in premature protein truncation. Both variants are therefore predicted to impair SLCO2A1 function through a loss-of-function mechanism, which is consistent with the known disease mechanism underlying PHO. Although RNA-based functional studies were not available to directly confirm the effect of c.940 + 1G>A on splicing, the clinical phenotype of the patient and the ACMG classification of both variants support their pathogenicity. Nevertheless, the lack of experimental validation remains a limitation of the present study. However, considering the frequency of variants, myelofibrosis has been observed exclusively in individuals with biallelic loss-of-function variants, whereas family members carrying only one monoallelic variant are typically asymptomatic or only mildly affected, suggesting that more severe functional impairment may predispose to hematologic involvement (4, 12, 13). Clinical and genetic findings in PHO patients with SLCO2A1 mutations and myelofibrosis are summarized in **Table 1**.

**Table 1:** Clinical and genetic findings in PHO patients with concurrent myelofibrosis and SLCO2A1 mutations.

Patient	Case	Origin	Gender	Age of diagnosis	SLCO2A1 mutations	Zygosity	Inheritance	Consanguineous marriage	Testing method	Myelofibrosis	Anemia	Other manifestations and comments	Outcome
1	Current case	Chinese	Male	45	c.940 + 1G>A (p.)? + c.440G>A (p.Trp147*)	Compound heterozygous	AR	N	WES	Y	Y (52g/L)	Clubbing, pachydermia, periosteal reaction	Mild fatigue In follow-up
2	Yousaf M 2022 (13)	Pakistani	Male	21	c.664G>A (p.Gly222Arg)	Homozygous	AR	Y	N/A	Y	Y (transfusion dependency)	Clubbing, pachydermia, periosteal reaction	EPO and COX-2 inhibitors reduce transfusion
3	Yousaf M 2022 (13)	Pakistani	Female	15	c.664G>A (p.Gly222Arg)	Homozygous	AR	Y	N/A	N/A	Y (transfusion dependency)	Sibling of patient 2 Clubbing, pachydermia, periosteal reaction	N/A
4	Yousaf M 2022 (13)	Pakistani	Male	5	c.664G>A (p.Gly222Arg)	Homozygous	AR	Y	N/A	N/A	N/A	Sibling of patient 2 Asymptomatic	N/A
5	Wang Q 2019 (8)	Chinese	Male	28	c.1807C>T (p.Arg603*)	Homozygous	N/A	N/A	N/A	Y	Y (70-80g/L)	Clubbing, pachydermia, periosteal reaction, arthralgia, intestinal lesion (diarrhea, hematochezia, intestinal obstruction)	COX-2 inhibitors, NSAIDs, enterectomy improved all symptoms
6	Diggle CP 2012 (12)	Columbian	Male	49	c.1259G>T (p.Cys420Phe)	Homozygous	N/A	Y	WES	Y	Y	PHO onset at age 19, pancytopenia and myelofibrosis diagnosed at age 49	N/A
7	Diggle CP 2012 (12)	North African	Male	16	c.664G>A (p.Gly222Arg)	Homozygous	N/A	Y	N/A	Y	Y	PHO diagnosed at age 17 and myelofibrosis at age 32. Hyperplastic gastrophy	N/A
8	Diggle CP 2012 (12)	North African	Male	17	c.253A>T (p.Ile85Phe)	Homozygous	N/A	Y	N/A	Y	Y	PHO diagnosed at age 16 and myelofibrosis at age 21. Affected brother not studied.	N/A
9	Diggle CP 2012 (12)	Turkish	Male	17	c.542G>A (p.Gly181Asp)	Homozygous	N/A	Y	N/A	Y	Y (36g/L)	Pancytopenia	N/A
10	Diggle CP 2012 (12)	Turkish	Male	21	c.542G>C (p.Gly181Ala)	Homozygous	N/A	Y	N/A	Y	Y	PHO diagnosed at age 21, anemia and myelofibrosis at age 24, renal amyloidosis at age 27	N/A

All individuals carrying biallelic SLCO2A1 mutations and reported to have myelofibrosis and/or anemia are included, together with relevant family members for whom clinical and genetic information was available. Variant annotation is based on SLCO2A1 reference transcript NM_005630.3. Y, Yes; N, No; N/A, not applicable or not available; WES, whole exome sequencing; AR, autosomal recessive; AD, autosomal dominant.

Management options and genetic counseling were discussed with the patient following diagnosis. Nonsteroidal anti-inflammatory drugs (NSAIDs), especially selective COX-2 inhibitors, have been reported to improve skin symptoms (2) and some gastrointestinal manifestations including gastric mucosa hyperplasia by reducing prostaglandin levels and decreasing IL-6, TNF-α and RANKL expression (14), which suggests that selective COX-2 inhibitors may play a potentially important role in the treatment of PHO. However, PHO is characterized by heterogeneous and variable clinical manifestations with a broad spectrum of severity, and the optimal treatment may vary depending on predominant clinical phenotype. In the present case, the primary concern was severe anemia secondary to myelofibrosis and gastrointestinal bleeding rather than skin or articular symptoms. Moreover, the balance of potential benefits and risks of selective COX-2 inhibitors in high-risk patients with a history of gastrointestinal bleeding or ongoing ulcer lesions remains insufficiently defined. Therefore, COX-2-targeted therapy was not administered. Meanwhile, the patient declined further medical intervention to avoid additional medical visits. As he has no further reproductive plans, future genetic counseling is recommended for his children (aged 19 and 9). Preimplantation genetic testing may reduce the risk of transmitting pathogenic variants to the next generations.

## Conclusion

This case describes a PHO patient with compound heterozygous mutations in SLCO2A1. Hematologic evidence suggests that myelofibrosis is more likely to occur in patients with biallelic SLCO2A1 mutations. Further studies are needed to better understand the underlying mechanisms and to develop clearer guidance for diagnosis and management.

## Data Availability

The original contributions presented in the study are included in the article/supplementary material. Further inquiries can be directed to the corresponding author.

## References

[1] Harbison JB Nice CM . Familial pachydermoperiostosis presenting as an acromegaly-like syndrome. Am J Roentgenol Radium Ther Nucl Med. (1971) 112:532–6. doi: 10.2214/ajr.112.3.532 5570364

[2] Lu Q Xu Y Zhang Z Li S Zhang Z . Primary hypertrophic osteoarthropathy: Genetics, clinical features and management. Front Endocrinol (Lausanne). (2023) 14:1235040. doi: 10.3389/fendo.2023.1235040 37705574 PMC10497106

[3] Cai X Yang X Zhang P Dou Z Chen Z Zhu C . Distinct features of three clinical subtypes in 533 patients with primary hypertrophic osteoarthropathy. Orphanet J Rare Dis. (2025) 20:188. doi: 10.1186/s13023-025-03722-3 40251683 PMC12007382

[4] Li S Li Q Wang Q Chen D Li J . Primary hypertrophic osteoarthropathy with myelofibrosis and anemia: A case report and review of the literature. Int J Clin Exp Med. (2015) 8:1467–71. doi: 10.1007/s10067-025-07405-z 25785156 PMC4358611

[5] Uppal S Diggle CP Carr IM Fishwick CW Ahmed M Ibrahim GH . Mutations in 15-hydroxyprostaglandin dehydrogenase cause primary hypertrophic osteoarthropathy. Nat Genet. (2008) 40:789–93. doi: 10.1038/ng.153 18500342

[6] Zhang Z Xia W He J Zhang Z Ke Y Yue H . Exome sequencing identifies SLCO2A1 mutations as a cause of primary hypertrophic osteoarthropathy. Am J Hum Genet. (2012) 90:125–32. doi: 10.1016/j.ajhg.2011.11.019 22197487 PMC3257902

[7] Yuan L Chen X Liu Z Wu D Lu J Bao G . Novel SLCO2A1 mutations cause gender-differentiated pachydermoperiostosis. Endocr Connect. (2018) 7:1116–28. doi: 10.1530/EC-18-0326 30352415 PMC6223238

[8] Wang Q Li YH Lin GL Li Y Zhou WX Qian JM . Primary hypertrophic osteoarthropathy related gastrointestinal complication has distinctive clinical and pathological characteristics: Two case reports and review of the literature. Orphanet J Rare Dis. (2019) 14:297. doi: 10.1186/s13023-019-1247-6 31878983 PMC6933916

[9] Hoggatt J Singh P Sampath J Pelus LM . Prostaglandin E2 enhances hematopoietic stem cell homing, survival, and proliferation. Blood. (2009) 113:5444–55. doi: 10.1182/blood-2009-01-201335 PMC2689046 19324903

[10] Harada S Rodan GA Shamir D Weinreb M Keila S . Induction of vascular endothelial growth factor expression by prostaglandin E2 and E1 in osteoblasts. J Clin Invest. (1994) 93:2490–6. doi: 10.1172/JCI117258 8200985 PMC294462

[11] Elwakeel E Brüne B Weigert A . PGE2 in fibrosis and cancer: Insights into fibroblast activation. Prostaglandins Other Lipid Mediat. (2019) 144:106339. doi: 10.1016/j.prostaglandins.2019.106339 31100473

[12] Diggle CP Parry DA Logan CV Laissue P Rivera C Restrepo CM . Prostaglandin transporter mutations cause pachydermoperiostosis with myelofibrosis. Hum Mutat. (2012) 33:1175–81. doi: 10.1002/humu.22111 22553128

[13] Yousaf M Khan R Akram Z Chaudhry QU Iftikhar R . Primary hypertrophic osteoarthropathy with myelofibrosis. Cureus. (2022) 14:e30108. doi: 10.7759/cureus.30108 36381760 PMC9643122

[14] Huang H Wang Y Cao Y Wu B Li Y Fan L . Interleukin-6, tumor necrosis factor-alpha and receptor activator of nuclear factor kappa ligand are elevated in hypertrophic gastric mucosa of pachydermoperiostosis. Sci Rep. (2017) 7:9686. doi: 10.1038/s41598-017-09671-7 28851954 PMC5574921

